# Nucleo-Cytoplasmic Trafficking of TRIM8, a Novel Oncogene, Is Involved in Positive Regulation of TNF Induced NF-κB Pathway

**DOI:** 10.1371/journal.pone.0048662

**Published:** 2012-11-12

**Authors:** Dhanendra Tomar, Lakshmi Sripada, Paresh Prajapati, Rochika Singh, Arun Kumar Singh, Rajesh Singh

**Affiliations:** Department of Cell Biology, School of Biological Sciences and Biotechnology, Indian Institute of Advanced Research, Gandhinagar, India; National Cancer Institute (INCA), Brazil

## Abstract

TNF induced nuclear factor kappa B (NF-κB) is one of the central signaling pathways that plays a critical role in carcinogenesis and inflammatory diseases. Post-translational modification through ubiquitin plays important role in the regulation of this pathway. In the current study, we investigated the role of TRIM8, member of RING family ubiquitin ligase in regulation of NF-κB pathway. We observed that TRIM8 positively regulates TNF induced NF-κB pathway. Different domains of TRIM8 showed discrete functions at the different steps in regulation of TNF induced NF-κB pathway. Ubiquitin ligase activity of TRIM8 is essential for regulation of NF-κB activation in both cytoplasm as well as nucleus. TRIM8 negates PIAS3 mediated negative repression of NF-κB at p65 by inducing translocation of PIAS3 from nucleus to cytoplasm as well as its turnover. TNF induces translocation of TRIM8 from nucleus to cytoplasm, which positively regulates NF-κB. The cytoplasmic translocation of TRIM8 is essential for TNF induced NF-κB but not for p65 mediated NF-κB regulation. TRIM8 also enhanced the clonogenic and migration ability of cells by modulating NF-κB. The further study will help to understand the role of TRIM8 in inflammation and cancer.

## Introduction

NF-κB is an inducible transcription factor and known to be involved in various physiological and pathological conditions [1]. The activation of NF-κB leads to transcription of the genes regulating cell cycle, immune response and cell death [2], [3], [4]. The dysregulation of this pathway has been observed in many cancer, neurodegeneration, skeletal abnormalities, autoimmune diseases and metabolic disorders [5], [6]. It is regulated by variety of patho-physiological stimuli however generates a unique response for particular stimuli. Tumor necrosis factor alpha (TNF-alpha) induced NF-κB affects many cellular functions including growth, differentiation, inflammation, immune responses and apoptosis through regulation of NF-κB pathway [7], [8]. Hence, TNF induced NF-κB pathway has been a focus of investigation for last several years [1], [2], [3], [6], [9].

Posttranslational modification of proteins by ubiquitin has been known to play important role in regulation of NF-κB pathway. The process of ubiquitination is achieved by the sequential action of three enzymes: E1 (Ub activating enzyme), E2 (Ub conjugating enzyme), E3 (Ub ligases). Recent evidences suggest that all the enzymes of this pathway have unique role in regulation of NF-κB pathway [9]. The terminal enzyme E3, transfers Ub from the E2 to a lysine residue on a substrate protein, resulting in an isopeptide bond formation between the lysine of substrate and the C-terminal glycine of Ub. E3 ligases provide specificity to the pathway as they recognize the substrates, interact with definite E2 to determine the topology of ubiquitination. The role of ubiquitination in regulation of NF-κB pathway is evolving and several untraditional roles have been discovered like stabilization of proteins through K63 linkages, formation of linear ubiquitin (Ub) chains [9], [10], [11]. E3 ligases may be critical in many of these unique linkages of target proteins through ubiquitin.

The binding of TNF to its cognate receptor TNFR1, leads to recruitment of several ubiquitin ligases like TRAF2, cIAP1 and cIAP2 and kinase RIP1 [12]. These ligases are either auto-ubiquitinated and/or ubiquitinate other substrates to activate downstream central kinase complex (IKKα/IKKβ/IKKγ), resulting in translocation of NF-κB to nucleus. Interestingly, it has been recently observed that two proteins known as heme-oxidised IRP1 Ub ligase-1 (HOIL-1) and the HOIL-1-interacting protein (HOIP), which together constitute Linear Ubiquitin-chain Assembly complex (LUBAC) is recruited to TNFR1 in a ligand-dependent manner [13]. This complex forms linear ubiquitin chains that regulate activation of NF-κB [14]. These evidences suggest that there is additional level of complexity in regulation of NF-κB activation through recruitment of different ubiquitin ligases in cell type and stimulus specific conditions.

TRIM/RBCC belongs to subset of RING family of Ub E3 ligases, consisting of N terminus **R**ING domain, **B**-Box and coiled-coil (**CC**) domain (**RBCC**) [15]. TRIM proteins have been implicated in a variety of processes like development, differentiation and innate immunity. We have initiated study to understand the role of TRIM family proteins in regulation of stress signaling pathways [16]. The role of TRIM family proteins in regulation of NF-κB is emerging [17], [18], [19], [20]. Recently it has been observed that TRIM8 (Tripartite motif containing protein 8) modulates the activity of transcription factors like SOCS-1 and STAT3 [21], [22]. In the current study, we report that TRIM8 positively regulates TNF induced NF-κB activation at p65 level by inducing the translocation of PIAS3 (Protein Inhibitor of Activated STAT-3) from nucleus to cytoplasm. Nucleo-cytoplasmic translocation of TRIM8 is important for positive regulation of NF-κB activation. TRIM8 also regulates clonogenic and migration ability of the cells through NF-κB pathway.

## Experimental Procedures

### Cells and reagents

HEK293, MCF7, HeLa and all other cell lines were grown at 37°C, 5%CO_2_ in Dulbecco's Modified Eagle's Medium (DMEM, Lonza, Switzerland) supplemented with 10% (v/v) heat-inactivated fetal bovine serum (FBS, Lonza, Switzerland), 1% penicillin, streptomycin, and amphotericin B (PSA) antibiotic mixture (Lonza, Switzerland). TNFα, leptomycin B and cycloheximide were purchased from Sigma Aldrich, USA. The primary antibodies used were- Anti-Flag-HRP, anti-PIAS3 (Sigma, USA), anti-HA peroxidase (Roche, Germany), mouse monoclonal against GFP (Clontech Laboratories, Inc. CA, USA), rabbit polyclonal against ATG7 (Abcam, USA), rabbit polyclonal against β-Actin (Abcam, USA). HRP-conjugated anti-rabbit and anti-mouse antibodies (Open Biosystems) were used. Full length TRIM8 was provided by Dr. W. Mothes (Section of Microbial Pathogenesis, Yale University School of Medicine, USA) [23]; HA-TRIM8 by Dr. S. Hatakeyama (Department of Biochemistry, Hokkaido University Graduate School of Medicine, Japan) [21]; TRIM8-GFP, TRIM8-ΔRING-GFP, TRIM8-ΔB1-GFP, TRIM8-ΔCC-GFP, TRIM8-ΔC-terminal-GFP by Dr. G. Meroni (Cluster in Biomedicine, AREA Science Park, Trieste, Italy) [15]. FLAG-PIAS3 by Dr. Y. Tsuji (Department of Environmental and Molecular Toxicology, North Carolina State University, USA) [24]; PIAS3-GFP by Prof. E. Razin (Department of Biochemistry, Hebrew University-Hadassah Medical School, Jerusalem, Israel) [25]; Flag-TAK1 by Dr. Y. Y. Wang (College of Life Sciences, Wuhan University, China) [26].

### NF-κB Luciferase Assay

To assess NF-κB activity, HEK293 cells were seeded at density of 1.5×10^5^ cells per well in 24 well plate. The cells were transfected with NF-κB luciferase reporter plasmid together with indicated expression vector using standard calcium phosphate method [27]. Renilla luciferase expressing plasmid was used for normalizing transfection efficiencies. The total amount of DNA (0.5 µg) was kept constant by inclusion of vector. After 24 hours of transfection, the cells were treated with TNFα (10 ng/ml) for 10 hours. The luciferase activity was determined using Dual-Glo® luciferase assay system according to the manufacturer's instructions (Promega) and measured with a Centro LB 960 Luminometer (Berthold Technologies). Assays were performed in triplicate and repeated three times. The activation of NF-κB in HeLa cells were also monitored in similar way however, 1.25×10^5^ cells/well was plated.

### RT-PCR analysis

The expression of TRIM8 in different cell lines and knockdown in HeLa cells were analyzed by RT-PCR. Total RNA was extracted from the cells using GenElute Mammalian Total RNA Miniprep Kit (Sigma Aldrich, USA) according to the manufacturer's protocol. One step RT-PCRs were performed using BluePrint One Step RT-PCR Kit (Takara, Japan) with TRIM8 specific primers: forward, 5′-CGCGGATCCATGCTAGAAGGCCCCTTC-3′ and reverse, 5′-CCGCTCGAGGCTCGTCACGTAGTGTTT-3′; and β-Actin: forward, 5′- TCGTGCGTGACATTAAGGGG-3′ and reverse, 5′- GTACTTGCGCTCAGGAGGAG-3′. The cycling conditions were 42°C for 45 min, 95°C for 10 min, followed by 35 cycles at 95°C for 30 s, at 48°C for 30 s, and at 72°C for 90 s and a final extension at 72°C for 10 min. The PCR products were analyzed by electrophoresis on 1% agarose gel containing ethidium bromide and photographed by CN-08 Infinity gel imaging system (Vilber Lourmat, France).

### Fluorescence Microscopy

#### p65-GFP translocation assay

HEK293 cells were plated at density of 1.5×10^5^ cells per well in 24 well plate and co-transfected with p65-GFP, TRIM8 and vector. After 24 hours of transfection, the cells were treated with TNFα (10 ng/ml) and localization of p65-GFP was monitored at different time interval using IX81 fluorescent microscope (Olympus, Japan). Images were analyzed by Image Pro Plus 6.1.0 software (Media Cybernetics, Inc. USA). Minimum 20 images and 150 cells were used for analysis.

#### Cellular localization of TRIM8 and PIAS3

To study the cellular localization of TRIM8 and PIAS3, HEK293 cells were plated at density of 1.5×10^5^ cells/well in 24 well plate on cover slip and transfected with indicated expression vector. After 24 hours of transfection, the cells were treated with indicated stimuli for specified time, fixed by 4% para-formaldehyde and counterstained with DAPI. The cells were monitored by Leica TCS SP5 II confocal microscope (Leica Microsystems, Germany). Images were captured, pseudo-coloured and analyzed by Leica LAS AF software. For time course study of nucleo-cytoplasmic translocation of TRIM8, cells were transfected with TRIM8-GFP and treated with TNFα for indicated time point. Nucleus was stained by Hoechst 33342 (Life Technologies, USA) and observed under 1X81 fluorescent microscope. Minimum 20 images and 150 cells were used for analysis.

### Nuclear fractionation and western blotting

The nuclear and cytosolic fractions were prepared as described previously [28] with minor modifications. HEK293 cells were plated at density of 1×10^6^ in 60-mm^2^ dish and transfected with HA-TRIM8. After 36 hours of transfection, the cells were treated with indicated chemical. After incubation, the cells were washed with chilled DPBS (GIBCO, Invitrogen, USA), resuspended in three-fold volume of buffer-A (10 mM HEPES buffer, pH 7.9, 0.1 mM EDTA, 10 mM KCl, 0.4% (v/v) NP40, 0.5 mM dithiothreitol (DTT), and 1 mM phenylmethylsulfonyl fluoride (PMSF) and incubated on ice for 30 min. Cell lysates were centrifuged at 15,000 g for 15 min and the resulting supernatant was collected as cytosolic fraction, re-centrifuged as above to remove nuclear remnants. Pellets were washed three times with buffer-A and resuspended in 2 fold volumes of ice cold buffer-B (20 mM HEPES buffer, pH 7.9, 400 mM NaCl, 1 mM EDTA, 1 mM DTT and 1 mM PMSF) and incubated in ice for 20 min. The lysate were centrifuged at 15000 g for 15 min to obtain nuclear fraction in the supernatant. The fractions were analyzed for the presence of indicated proteins using western blotting as described previously [16].

### Cellular proliferation assay using MTT

The cellular proliferation was analyzed by MTT assay [29]. MCF7 and HEK293 cells were seeded in 24-well plate at a density of 1.5×10^5^ cells/well. The cells were transfected with indicated gene of interest. After 24 hours of transfection, 20 µl of MTT solution (5 mg/ml) (Serva, Germany) was added to each well and incubated for 2 hours. After incubation, 400 µl of solubilization buffer (2% w/v SDS, 18.5% w/v formaldehyde) was added to dissolve the precipitate of purple colored formazan and color intensity was monitored using colorimetric microplate reader (BioTek Instruments, Inc. USA) at 595 nm wavelength.

### Colony formation assay

Clonogenic activity of cells was determined by colony forming assay described previously [30]. Briefly, the cells were transfected with indicated expression vector in 24 well plate via standard calcium phosphate method. After 24 hours of transfection, the cells were washed, counted and were plated at density of 3000 cells per 90 mm^2^ dish. Cells were further cultured for 7 days in standard conditions. The plates were washed with PBS, fixed with chilled methanol and stained with 0.2% crystal violet solution. Plates were imaged by CN-08 Infinity gel imaging system (Vilber Lourmat, France) and the colonies having more than 50 cells counted.

Plating efficiency is the ratio of the number of colonies to the number of cells seeded and calculated as following- [30].
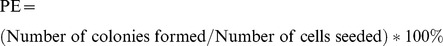



### Scratch Assay

In vitro scratch assay was performed to determine the migration ability of the cells [31]. HEK293 cells were seeded at density of 2.5×10^5^ cells per well in 12 well plate, transfected with TRIM8 and vector. After 24 hours of transfection, a vertical wound was created in the monolayer in each well using a sterile P200 micropipette tip (Axygen Inc., USA). The wells were washed and replaced with 1 ml of fresh DMEM. The first image of each scratch was acquired at zero time point using inverted phase contrast microscope (1X81 Olympus, Japan) at 10× magnification and plate was further incubated at 37°C, 5% CO_2_ for 24 hours. After every 12 hours, each scratch was examined and photographed at the same area. The images acquired for all wells were analyzed using TScratch program (www.cse-lab.ethz.ch). Rate of migration was analyzed by the T-scratch software which determines the open area at different time interval. The percentage open area in each condition was plotted using Graphpad Prism-5 software. The experiment was repeated three times.

### Statistical analysis

Data are shown as mean ± SEM for n observations. Comparisons of groups were performed using student t-test for repeated measurements to determine the levels of significance for each group. The experiments were performed minimum three times independently and p<0.05 was considered as statistically significant.

## Results

### TRIM8 positively regulates TNF induced NF-κB activation

**Figure 1:**
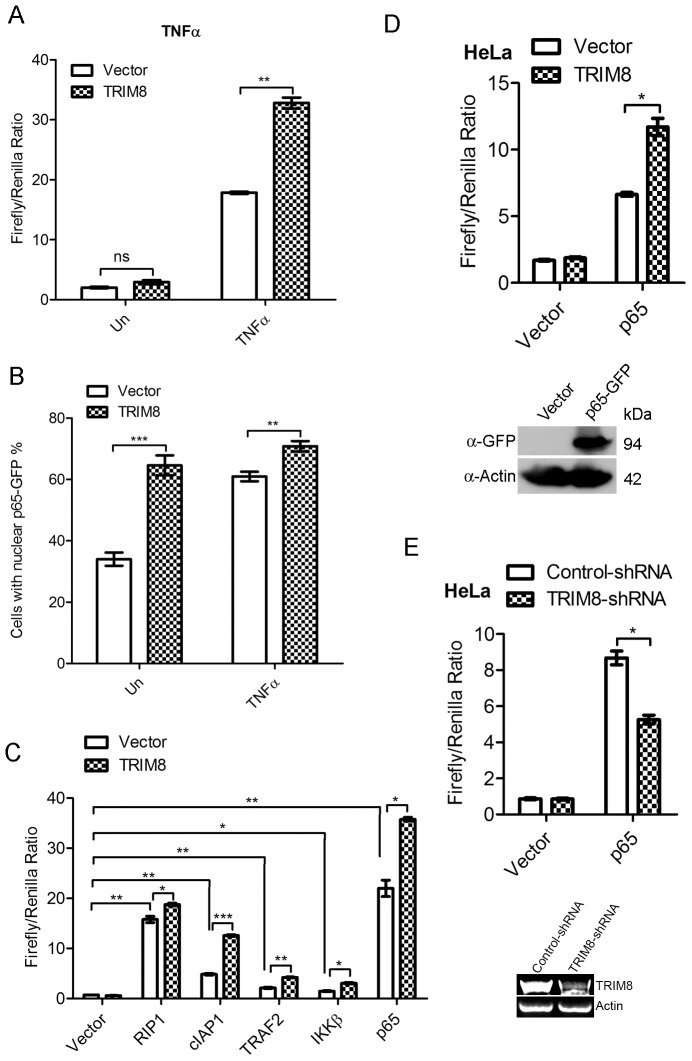
TRIM8 positively regulates TNFα induced NF-κB activation. (A) TRIM8 enhances TNFα induced NF-κB activation. HEK293 cells were transfected with TRIM8 and vector with NF-κB luciferase reporter constructs as described in materials and methods section. NF-κB activation was measured by Dual Glo luciferase assay. (B) TRIM8 induces p65 translocation to nucleus. Graphical representation of the numbers of cells with nuclear p65-GFP in TRIM8 and vector transfected cells as described in materials and method section. (C) TRIM8 positively regulates NF-κB at the level of p65. HEK293 cells were co-transfected with TRIM8, vector and specified gene. NF-κB activity was monitored by Dual Glo luciferase assay as described earlier. TRIM8 significantly enhances RIP1, cIAP1, TRAF2, IKKβ and p65 mediated NF-κB activation. (D) TRIM8 expression enhances p65 mediated NF-κB activation in HeLa cells. p65 overexpression was confirmed by western blotting. (E) TRIM8 knockdown suppresses p65 mediated NF-κB activation in HeLa cells. TRIM8 knockdown was validated by RT-PCR.

To elucidate the role of TRIM8 in NF-κB pathway, we co-transfected TRIM8 and vector with NF-κB reporter construct in HEK293 cells and monitored NF-κB activity. The ectopic expression of TRIM8 showed no significant change in NF-κB activation in normal condition. Interestingly, treatment of TRIM8 transfected cells with TNFα showed significantly enhanced NF-κB activation as compared to vector (Figure 1A). We monitored NF-κB activation at different time points after TNF treatment. TRIM8 transfected cells, treated with TNF showed enhanced NF-κB activity as compared to vector at all time points (Figure S1). NF-κB activation was also monitored using p65-GFP translocation assay. The expression of TRIM8 induces nuclear translocation of p65, which increased during TNFα treatment (Figure 1B). These observations suggested that TRIM8 acts as positive regulator of TNF induced NF-κB pathway.

To characterize the step regulated by TRIM8 in NF-κB pathway, we co-transfected TRIM8 with different genes (RIP1, cIAP1, TRAF2, IKKβ, RelA/p65) involved in regulation at different levels of NF-κB pathway. TRIM8 significantly increased RIP1, cIAP1, TRAF2, IKKβ mediated NF-κB activity (Figure 1C) as compared to control. To characterize the further downstream mechanism, TRIM8 was co-transfected with p65 and NF-κB activity was monitored. TRIM8 enhanced nearly 1.5 fold p65 mediated NF-κB activation in normal conditions (Figure 1C). To rule out the cell line specific action, the effect of TRIM8 on NF-κB activity was also studied in HeLa cells. The transfection of TRIM8 in HeLa cells, significantly enhanced p65 mediated NF-κB activity (Figure 1D). It has been reported earlier and our data suggest that TRIM8 is endogenously expressed in HeLa cells [21]. To determine the role of endogenous TRIM8 in regulation of NF-κB activity, TRIM8 was knockdown using shRNA and monitored NF-κB. The p65 mediated NF-κB activation significantly decreased in TRIM8-shRNA transfected cells (Figure 1E). p65 overexpression in HeLa was confirmed by western blotting and TRIM8 knockdown was validated by RT-PCR. These observations suggest that TRIM8 acts at p65 or downstream to positively regulate NF-κB activity.

### Discrete functions of domains of TRIM8 in regulation of TNF induced NF-κB activation

**Figure 2:**
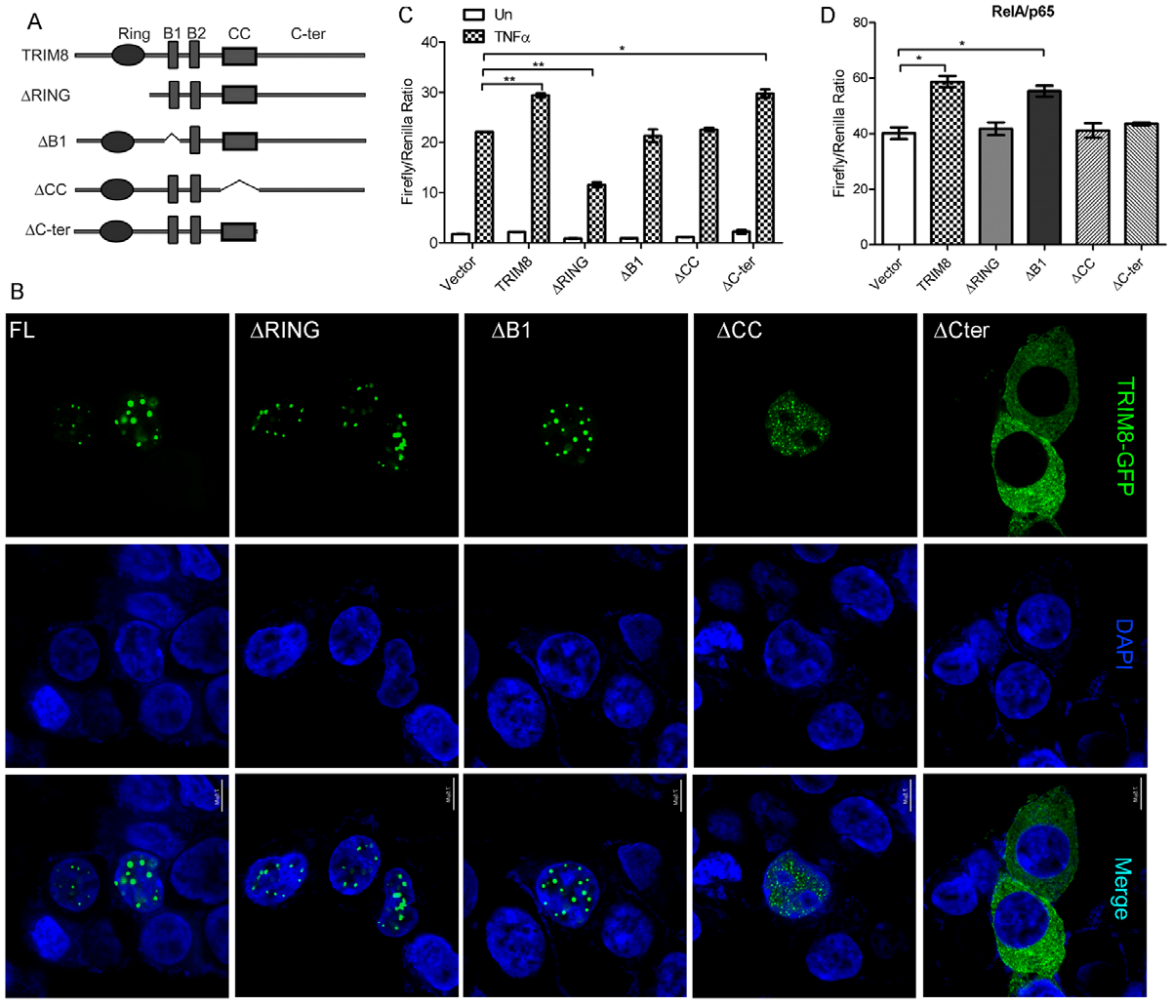
Cellular localization of different domains of TRIM8 and their role in NF-κB activation. (A) Schematic diagram showing TRIM8 domains and constructs used for transfection. (B) Cellular localization of TRIM8 deletion constructs. HeLa cells were plated on cover slip and transfected with GFP-tagged different deletion constructs of TRIM8. After 24 hours of transfection, the cells were fixed with 4% paraformaldehyde, stained with DAPI and visualized by confocal microscopy. Scale bar represent 7.5 µM. (C) RING domain is essential for positive regulation of TNF induced NF-κB activation. HEK293 was co-transfected with full length TRIM8, ΔRING, ΔB1, ΔCC, ΔC-ter, vector, treated with TNFα and NF-κB activation measured by Dual Glo luciferase assay. (D) RING, CC domain and C-terminal region of TRIM8 is essential for p65 mediated NF-κB activity. HEK293 was co-transfected with full length TRIM8, ΔRING, ΔB1, ΔCC, ΔC-ter, vector with p65-GFP and NF-κB activation measured by Dual Glo luciferase assay. Asterisk (*) indicates NF-κB activation significantly changed between groups: p value<0.05 for SEM of minimum three independent experiments.

TRIM8 is a multi-domain protein having N-terminal RING (R), B-box1 (B), coiled coil (CC) domain and C-terminal region (C-ter) (Figure 2A). Earlier studies suggest that different domains of TRIM family protein regulate cellular localization and higher order structures [32]. We studied the cellular localization of different deletions of TRIM8 using GFP fusion proteins. The full length (FL) TRIM8 showed predominantly nuclear localization and formed discrete foci (Figure 2B panel-1). The deletion of RING and B Box of TRIM8 did not change the nuclear localization, however, the size of foci decreased (Figure 2B panel 2, 3). The deletion of CC domain showed diffused nuclear staining and no discrete foci (Figure 2B panel 4). Interestingly the deletion of C terminus showed distinct cytoplasmic staining (Figure 2B panel 5). These results strongly suggest that C terminus is essential for its nuclear localization and CC domain involved in formation of its distinct discrete foci or higher order structures.

To characterize the domain of TRIM8 involved in NF-κB regulation, we co-transfected different deletion constructs of TRIM8 treated with TNFα and monitored NF-κB activity. The transfection of FL-TRIM8 showed increased NF-κB activation as compared to vector. The deletion of RING domain, results in suppression of NF-κB as compared to FL-TRIM8 and vector whereas deletion of B-box and CC domain results in abolishment of TRIM8 mediated positive regulation of NF-κB (Figure 2C). The deletion of C-terminal region, which is essential for their nuclear localization, does not affect the TRIM8 mediated positive regulation of TNF induced NF-κB activation (Figure 2C). These observations suggest that RING domain is essential for TRIM8 mediated positive regulation of TNF induced NF-κB activation whereas nuclear localization of TRIM8 is not essential.

The luciferase experiments showed that TRIM8 acts as positive regulator NF-κB activation at the level of p65 whereas results of domain deletion and their role in TNF regulated NF-κB showed that nuclear localization of TRIM8 is not essential for TNFα mediated positive regulation of NF-κB activation. Therefore, the question arose, how TRIM8 positively regulates p65 mediated NF-κB activation. To address this question, different deletion constructs of TRIM8 were co-transfected with p65 and NF-κB activity were monitored. The transfection of FL-TRIM8 showed increased NF-κB activation as compared to vector. The deletion of RING and CC domain results inhibited TRIM8 mediated positive regulation of NF-κB at the level of p65 (Figure 2D). Interestingly deletion of C terminus that abolished its nuclear localization, showed decreased p65 mediated NF-κB activation as compared to FL-TRIM8. These experiments suggest that TRIM8 may regulate TNF induced NF-κB at two steps, both in cytoplasm as well as nucleus. These results also suggest that different domains (RING, CC and C-terminal) may have discrete function in regulation of NF-κB.

### TRIM8 reverses PIAS3 mediated negative regulation of NF-κB

**Figure 3:**
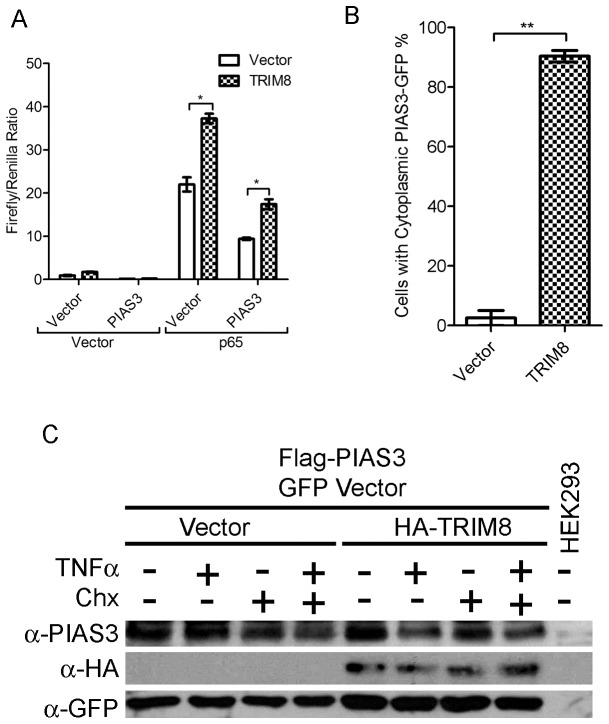
TRIM8 negates PIAS3 mediated NF-κB suppression. (A) TRIM8 recovers PIAS3 mediated NF-κB suppression. HEK293 cells were co-transfected with PIAS3, p65 and TRIM8 with vector and NF-κB luciferase reporter plasmids as described in materials and methods section. NF-κB activation was measured by Dual Glo luciferase assay. (B) Graphical representation of PIAS3 translocation. HeLa cells were transfected with HA-TRIM8, vector and PIAS3-GFP. After 24 hours of transfection, the cellular localization of PIAS3 was analyzed by confocal microscope. Asterisk (*) indicates data changes are statistically significant between groups: p value<0.05 for SEM of minimum three independent experiments. (C) TRIM8 increases PIAS3 turnover in the presence of TNF. HEK293 cells were transfected with HA-TRIM8, Flag-PIAS3 and GFP vector. After 36 hours of transfection, cells treated with indicated chemicals (TNFα and Cycloheximide- CHX) and probed by anti-PIAS3, anti-HA and anti-GFP antibody.

PIAS3 is known to negatively regulate NF-κB pathway via its interaction with p65 in the nucleus [33]. The direct role of TRIM8 in PIAS3 mediated negative regulation of NF-κB is not known. To check the role of TRIM8 in negative regulation of PIAS3 mediated NF-κB pathway, we co-transfected TRIM8 and p65 with PIAS3 in HEK293 cells and monitored NF-κB activity. PIAS3 expression downregulates p65 mediated NF-κB pathway was in consonance with earlier report [33]. Interestingly, the expression of TRIM8 significantly enhanced NF-κB activity in the presence of PIAS3. This clearly suggests that TRIM8 inhibited PIAS3 mediated negative regulation of NF-κB (Figure 3A). It has been earlier reported that TRIM8 may regulate the cellular localization of PIAS3 [21], therefore, cellular localization of PIAS3 was analyzed in the presence of TRIM8. In normal condition, PIAS3 was localized exclusively to the nucleus (Figure 3B). Interestingly, co expression of TRIM8 with PIAS3 showed translocation of PIAS3 to cytoplasm. The number of cells having cytoplasmic PIAS3 significantly increased (Figure 3B). As described in earlier sections that domain deletion and luciferase assay showed that RING domain is required TRIM8 mediated positive regulation of NF-κB at the level of p65. Therefore, we monitored turnover of PIAS3 in presence of TRIM8 during TNFα treatment. Western blotting experiment showed that TRIM8 overexpression decreases the level of 68 kDa protein band corresponding to PIAS3 in the presence of TNFα (Figure 3C). GFP was used as a transfection control in this experiment, which showed equal transfection efficiency in different conditions (Figure 3C). The level of p65 was also monitored in the presence of TRIM8, however no changes were observed (Figure S2). These observations suggest that TRIM8 mediated spatial regulation and turnover of PIAS3 may have important implication in regulation of NF-κB pathway at the level of p65.

### TRIM8 nuclear to cytoplasmic trafficking is essential for NF-κB regulation

**Figure 4:**
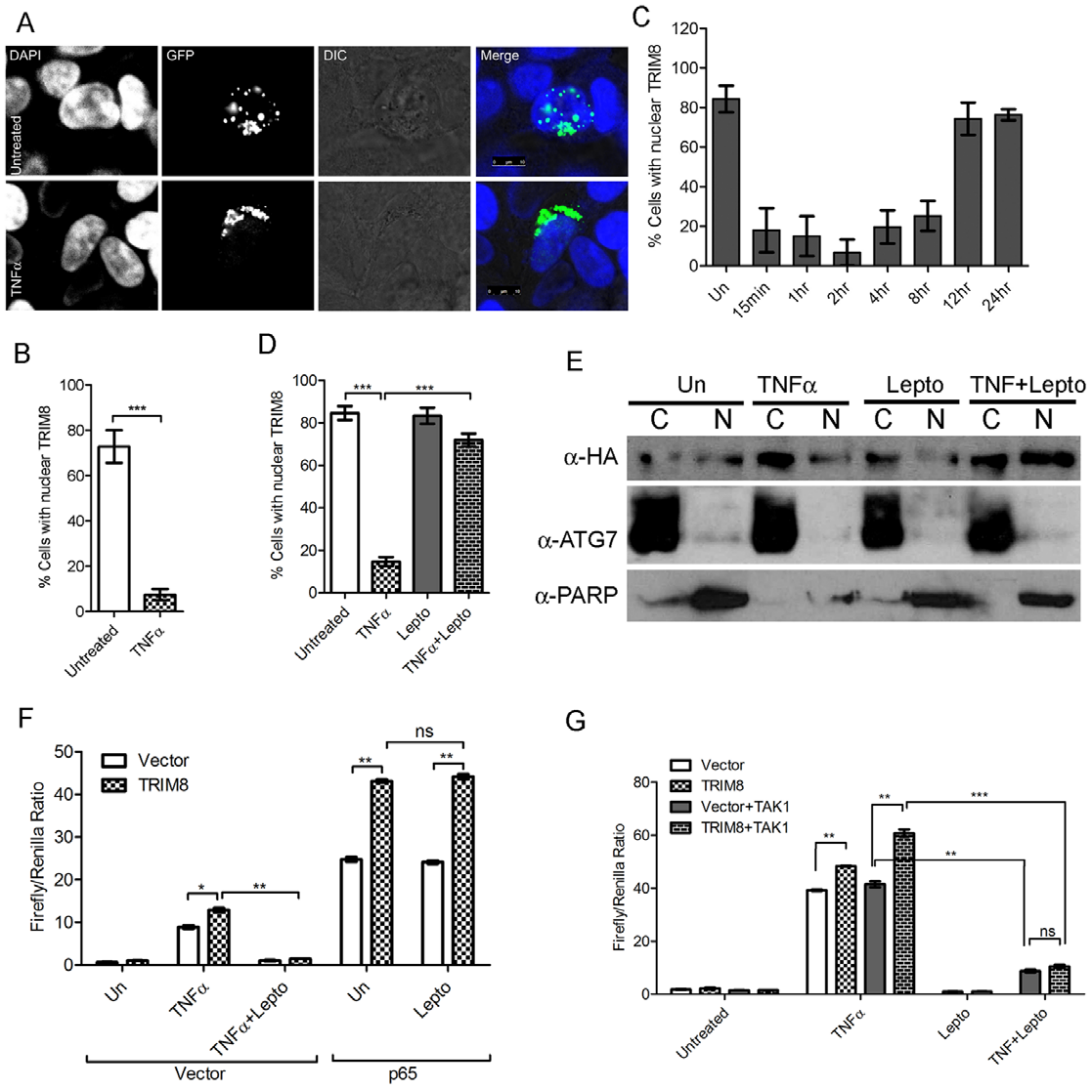
Nucleo-cytoplasmic translocation of TRIM8 is involved in regulation of NF-κB. (A) TRIM8 translocates to cytoplasm in the presence of TNFα. HEK293 cells were plated on cover slip and transfected with TRIM8-GFP. After 24 hours of transfection, the cells were treated with TNFα and fixed with 4% paraformaldehyde, stained with DAPI and visualized by confocal microscopy. Scale bar represent 7.5 µM. (B) Quantification of cells showing nuclear positive TRIM8-GFP in untreated and TNF treatment condition. (C) Time course analysis of nucleo-cytoplasmic localization of TRIM8 in response to TNF. Quantification of nuclear TRIM8-GFP positive cells in control and TNF treated conditions at indicated time points. (D) Leptomycin B suppresses TNF mediated cytoplasmic translocation of TRIM8. Quantification of cells showing nuclear TRIM8-GFP in indicated treatment conditions (Lepto = Leptomycin B). (E) Analysis of sub-cellular localization of TRIM8 in control and TNF treated cells. HEK293 cells were transfected with HA-TRIM8, nuclear and cytosolic fractions were isolated as described in materials and method. The fractions were analyzed by western blotting using indicated antibodies. (Lepto = Leptomycin B) (F) Leptomycin B suppresses TRIM8 mediated TNF induced NF-κB pathway. HEK293 was transfected with TRIM8, vector with or without p65, treated with Leptomycin B and NF-κB activation measured by Dual Glo luciferase assay. (Lepto = Leptomycin B) (G) Leptomycin B suppresses TAK1 induced NF-κB pathway. HEK293 was transfected with indicated constructs and NF-κB activation measured by Dual Glo luciferase assay (Lepto = Leptomycin B). Asterisk (*) indicates data changes are statistically significant between groups: p value<0.05 for SEM of minimum three independent experiments.

The results in the current study also suggest that nuclear as well as cytoplasmic localization of TRIM8 is required for NF-κB regulation (Figure 2C, D). During our manuscript preparation, Li *et. al.* observed that TRIM8 positively regulates NF-κB pathway by K-63 mediated polyubiquitination of TAK1 [26]. This report further strengthens our observations of TRIM8 mediated positive regulation of NF-κB. Our results in the current study and previous reports [15], [21] show that TRIM8 is nuclear protein however it is still not clear that how TRIM8 a nuclear protein, can regulate the ubiquitination of TAK1, a cytoplasmic protein. To answer this question, cellular localization of TRIM8 was monitored in absence/presence of TNFα. In untreated conditions, TRIM8 showed typical punctuate nuclear pattern. Interestingly, TRIM8 translocation from nucleus to cytoplasm was observed in the presence of TNFα (Figure 4A). The number of cells showing cytoplasmic TRIM8 significantly increased in the presence of TNFα (Figure 4B). Time course study for TRIM8 nucleo-cytoplasmic translocation was also done after TNF treatment. In response to TNF treatment, TRIM8 translocates to cytoplasm within 15 minute and re-translocates back to nucleus after 12 hours (Figure 4C) supporting our hypothesis that TRIM8 may regulate NF-κB both in cytoplasm as well as inside the nucleus. TRIM8 localization was also monitored in the presence of leptomycin B (nuclear export inhibitor). The treatment with leptomycin B significantly increased the number of cells showing nuclear positive TRIM8 in the presence of TNFα (Figure 4D). This observation was further confirmed by cellular fractionation and monitoring the presence of TRIM8 in nuclear and cytoplasmic fraction by western blotting. In normal condition, TRIM8 was observed in both nuclear and cytoplasmic fraction, whereas the levels of TRIM8 in cytoplasmic fraction increased in presence of TNF (Figure 4E). The inhibition of nuclear export by leptomycin B results in accumulation of TRIM8 in nuclear fraction (Figure 4E).

To confirm the role of this nucleo-cytoplasmic translocation of TRIM8 in regulation of NF-κB activation, we performed luciferase assay in the presence of leptomycin B. As observed in earlier experiments in this study, TRIM8 enhanced TNF induced NF-κB activation and was inhibited in the presence of leptomycin B (Figure 4F). Interestingly TRIM8 mediated positive regulation of p65 induced NF-κB is not affected by leptomycin B treatment (Figure 4F). As reported recently TAK1 is direct target of TRIM8, therefore we further checked the effect of nucleo-cytoplasmic translocation of TRIM8 on TAK1 mediated NF-κB activation. As observed previously TRIM8 enhanced TAK1 mediated TNF induced NF-κB activation (Figure 4G). Inhibition of TRIM8 nuclear export by leptomycin B results in inhibition of TAK1 mediated NF-κB activation (Figure 4G). These observations strongly suggest that nucleo-cytoplasmic translocation of TRIM8 in response to TNFα is important for positive regulation of NF-κB.

### TRIM8 knockdown decreases the clonogenic ability of MCF7

**Figure 5:**
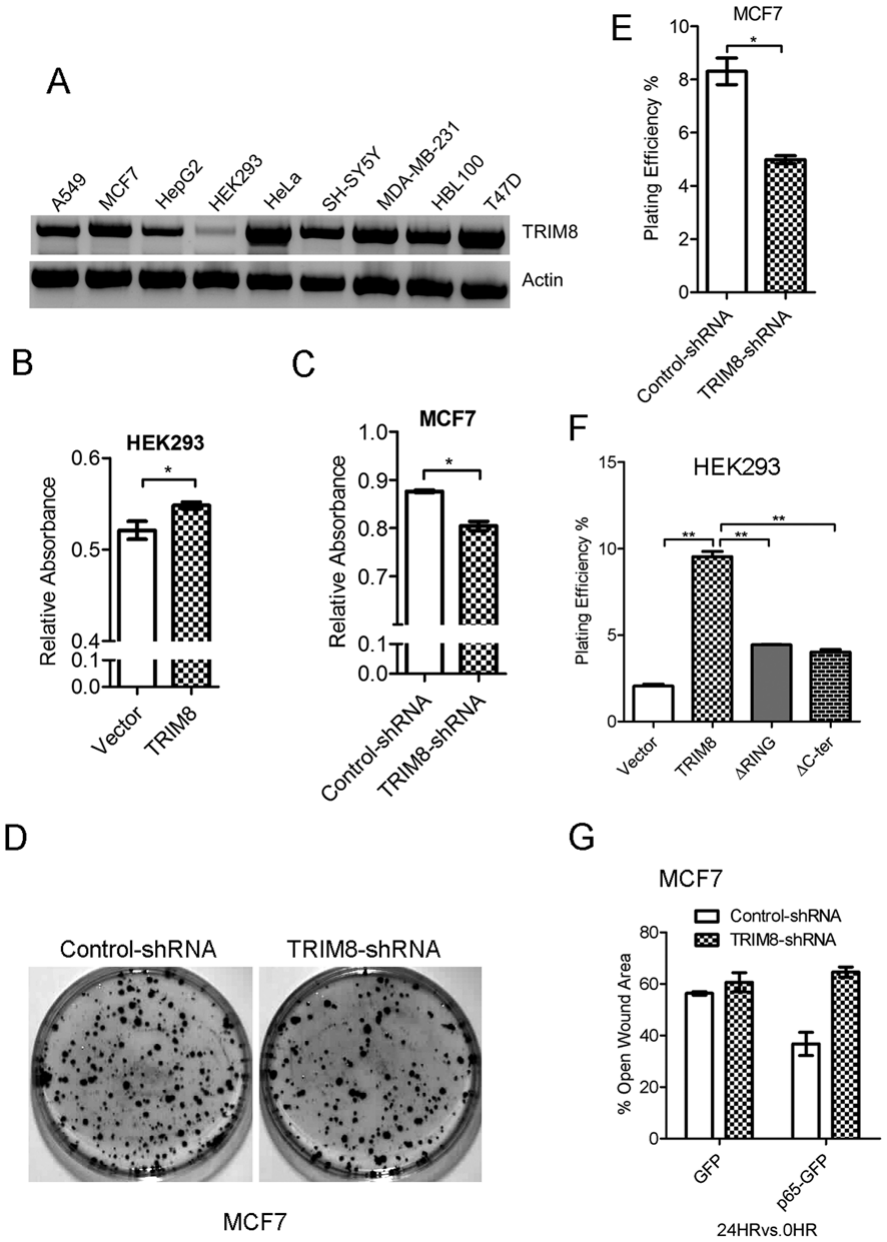
TRIM8 knockdown suppresses clonogenic and migration ability of the cells. (A) Analysis of TRIM8 expression in different cell lines. Total RNA was isolated from indicated cell lines and RT-PCR was performed as described in materials and method section. (B) TRIM8 overexpression increases proliferation of HEK293 cells. The cells were transfected with vector and TRIM8 and cellular proliferation was monitored by MTT assay. (C) TRIM8 knockdown decreases proliferation of MCF7 cells. The cells were transfected with vector and TRIM8 and cell proliferation was cellular monitored by MTT assay (D) TRIM8 knockdown suppresses clonogenic ability of MCF7 cells. The cells were transfected with TRIM8-shRNA and control-shRNA, clonogenic activity assessed as described in method section. (E) TRIM8 knockdown decreases plating efficiency of MCF7 cells. Graphical representation of plating efficiency of TRIM8-shRNA and control-shRNA transfected cells. (F) RING and C-ter region of TRIM8 is essential for regulation of clonogenic ability of MCF7 cells. Graphical representation of plating efficiency of FL-TRIM8, ΔRING-TRIM8, ΔC-ter-TRIM8 and vector transfected HEK293 cells. (G) TRIM8 is essential for p65 regulated migration ability of MCF7 cells. The cells were co-transfected with control and TRIM8 shRNA, with vector and p65 and scratch assay performed. Statistical analysis for open wound area formed in p65 overexpression and TRIM8 knockdown cells. Asterisk (*) indicates data changes are statistically significant between groups: p value<0.05 for SEM of minimum three independent experiments.

The evidence of positive regulation of NF-κB observed here and previous studies suggest that TRIM8 may act as oncogene [21] however, experimental evidences at cellular level is still lacking to support this hypothesis. We studied the expression of TRIM8 in various human cell lines and observed high expression in MCF7 and low expression in HEK293 cells (Figure 5A). As HEK293 have lowest expression of TRIM8, we firstly checked if TRIM8 overexpression regulates cell proliferation of HEK293 cells. The HEK293 cells were transfected with TRIM8 and cellular proliferation was assessed by MTT assay. Interestingly, the expression of TRIM8 in HEK293, significantly increased the cellular proliferation (Figure 5B). The knockdown of TRIM8 in MCF7 showed decreased cell proliferation as compared to control (Figure 5C). The overexpression of TRIM8 in MCF7 does not have any effect on cell proliferation (data not shown).

We have also studied the effect of TRIM8 on clonogenic ability of the cells. MCF7 cells were used for confirming the role of TRIM8 in clonogenic ability of the cells. MCF7 cells were transfected with TRIM8-shRNA and clonogenic (or colony formation) assay performed. The clonogenic ability (shown as plating efficiency) of TRIM8-shRNA transfected MCF7 cells significantly decreased as compared to control-shRNA (Figure 5D, E). This observation was also confirmed in HEK293 where the endogenous level of TRIM8 is low. The overexpression of TRIM8 in HEK-293 significantly increased the clonogenic ability of the cells (Figure S3). To determine the domain involved in regulation of clonogenic ability of cells, FL, ΔRING and ΔC-ter TRIM8 were transfected in HEK293 cells and clonogenic ability monitored. The expression of ΔRING and ΔC-ter showed significantly decreased plating efficiency as compared to FL-TRIM8 (Figure 5F, S4). This suggests that TRIM8 induced NF-κB and its nuclear localization is essential for clonogenic ability of the cells.

We also performed wound healing assay to assess the migration ability of the cells in presence/absence of TRIM8. HEK293 cells transfected with TRIM8 showed increased migration as the percentage of open wound area decreased as compared to control (data not shown). To confirm the role of TRIM8 mediated regulation of NF-κB in migration ability of the cells, the scratch assay was performed in the presence of p65. Knockdown of TRIM8 in MCF7 cells showed no effect on migration ability whereas p65 overexpression increased the migration ability as percentage open would area decreased (Figure 5G). The expression of p65 showed decreased migration ability of the cells in TRIM8 knockdown as compared to control-shRNA (Figure 5G). This clearly suggests that TRIM8 mediated positive regulation of NF-κB at the level of p65 is essential for migration ability of the cells.

## Discussion

TNF is a pleiotropic cytokine and plays critical role in inflammation, metabolism, tumorigenesis and other pathological conditions [34], [35]. TNF induced NF-κB activation is critical regulator of cell survival and death, having implications in many physiological and pathological conditions including cancer. Further understanding of NF-κB regulation is critical for selective modulation of this pathway in given pathological conditions. Activation of NF-kB is regulated by several ubiquitin ligases starting from the assembly of complex at TNF receptor, p65 translcoation and binding to promoter of the specific gene in nucleus [9], [14], [36]. In recent studies, nuclear ubiquitin E3 ligases have been identified that ubiquitinate p65 and induce proteosomal degradation in inflammatory conditions, thus terminating the response [37]. In the current study, TRIM8, a nuclear E3 ligase, was found to be positive regulator of TNF induced NF-kB pathway. In normal condition, this protein has neglible effect on NF-kB pathway. This may be due to high turnover of TRIM8 as observed previously [21]. We also observed accumulation of polyubiquitinated form of TRIM8 in the presence of inhibitor of proteasome pathway (data not shown). TRIM8 may get stabilized in the presence of TNF and positively regulates NF-κB.

TRIM8 has three characterstic domains: RING, B-box and coiled-coil (CC) domain. The experiments here suggest that RING domain responsible for ubiquitin ligase activity is important TNF induced NF-κB activation. The results of luciferase assays suggest that TRIM8 acts at the level of p65 or downstream to positively regulate TNF induced NF-κB pathway. It has been previously observed that PIAS3 interacts with p65 in nucleus, prevents binding to its cognate sites and inhibits NF-κB activation [12]. The evidences here suggests that TRIM8 induces translocation of PIAS3 from nucleus to cytosol, hence p65 becomes free to activate NF-κB responsive genes. This is also supported by our observation that deletion of C terminal region of TRIM8 leading to exclusive cytoplasmic localization and has no effect on p65 mediated NF-κB. This strongly suggest that nuclear retention of TRIM8 is important for positive regulation of NF-κB pathway at the level of p65. The current and previous evidences [21] suggest that TRIM8 may promote PIAS3 ubiquitnation via its ligase activity, translocation to cytoplasm and degradation through proteasome. Similarly TRIM proteins are known to form higher order structures or bodies [32]. The evidences from current study, suggest that CC domain regulate the formation of higher order structures or bodies, that may help in assembling the signalosome for regulation of NF-κB. The evidences here demonstrate that different domains of TRIM8 have unique function in regulation of TNF induced NF-kB pathway.

During our manuscript preparation Li et. al. reported that TRIM8 positively regulates NF-κB activation by TAK1 ubiquitination [26]. TAK1 is known to be cytoplasmic protein therefore the question of TRIM8 a nuclear protein, ubiquitinating TAK1 a cytoplasmic protein, is not understood. The analysis of cellular localization of TRIM8 strongly suggests that TRIM8 is translocated to cytoplasm in response to TNF. This translocation is inhibited by nuclear transport inhibitor that negatively regualates NF-κB activation. This suggests that TRIM8 acts at two subcellular sites in regulation of NF-κB pathway. In resposne to TNF it translocate to cytoplasm thus activating TAK1 and may facilitate p65 translocation. TRIM8 again translocate back to nucleus where it may act on PIAS3. This hypothesis is strongly supported by monitoring the time course of subcellular localization of TRIM8 in the presence of TNF. In nucleus it may exclude PIAS3 from nucleus and p65 may become free to activate NF-κB. It is also possible that TRIM8 may act as carrier protein for PIAS3 translocation and degradation resulting in NF-κB activation. These interesting hypothesis needs further experimental evidences to confirm multiple role of TRIM8 in NF-κB pathway.

The association between inflammation and cancer has been known for long time [17]. TNF is one of the pro-inflammatory cytokines that is constitutively present in tumor microenvoirnments and regulates various steps of tumorigenesis [38]. In the current study we clearly observed that TRIM8 positively regulates TNF mediated NF-κB activation suggesting that it may activate postive feedback loop that may be crucial for tumorigenesis. This hypothesis is further supported by the loss of expression of PIAS3, the target of TRIM8, in many tumors including human gastric carcinoma [39] and glioblastoma multiforme tumors [40]. The observation of TRIM8 mediated increase in cell motlity and clonogenic ability further supports that TRIM8 may act as tumor promoter in specific conditions. The increase of TRIM8 mediated cell motility further strengthen our hypothesis of role of TRIM8 mediated PAIS3 translocation to cytoplasm where it it may SUMOylate Rac1 which may stimulate lamellipodia, cell migration and invasion as recently observed [41]. This hypothesis is of further relevance as recently it has been observed that TRIM8 promotes anchorage independent growth [21].

The current study provided experimental evidence that TRIM8 positively regulates TNF induced NF-κB. This study raised several interesting questions if TRIM8 is autoubiquitinated and degraded in normal condition or stablized during inflammatory conditions. TNF induced nucleo-cytoplasmic translocation of TRIM8 is having implication is several cellular processes like NF-κB activation, PIAS3 translocation and cell migration. These various possiblites needs further study and may provide important insights into the role of TRIM8 and establshing linkages between inflammation and tumorigenesis.

## Supporting Information

Figure S1 **TRIM8 positively regulates TNF induced NF-κB.** HEK293 cells were transfected with TRIM8 and vector; treated with TNFα (10 ng/ml) for different time interval and NF-κB activation measured by Dual Glo luciferase assay. Asterisk (*) indicates p value<0.05 for SEM of minimum three independent experiments.
(TIF)

Figure S2 **TRIM8 expression has no effect on p65 protein turnover.** HEK293 cells were co-transfected with p65, TRIM8 and vector control. After 24 hours of transfection, the cells were treated with indicated chemical and incubated for 8 hours. The levels of different proteins were analyzed by western blotting using specific antibodies. HEK293 cell lysate was also loaded for negative control of HA-TRIM8 and p65-GFP.
(TIF)

Figure S3 **TRIM8 overexpression increases clonogenic ability of HEK293 cells.** TRIM8 and vector were transfected in HEK293 cells and clonogenic ability analyzed as described in materials and method section.
(TIF)

Figure S4 **RING domain and C-terminal region of TRIM8 is essential in regulation of clonogenic ability of cells.** FL-TRIM8, ΔRING, ΔC-ter and vector were transfected in HEK293 cells and clonogenic ability analyzed as described in materials and method section.
(TIF)
