# Supplementary figures and images for: Nucleo-Cytoplasmic Trafficking of TRIM8, a Novel Oncogene, Is Involved in Positive Regulation of TNF Induced NF-κB Pathway
Source: PLoS One. 2012 Nov 12;7(11):e48662. doi: 10.1371/journal.pone.0048662 (PMC3495970; doi:10.1371/journal.pone.0048662)

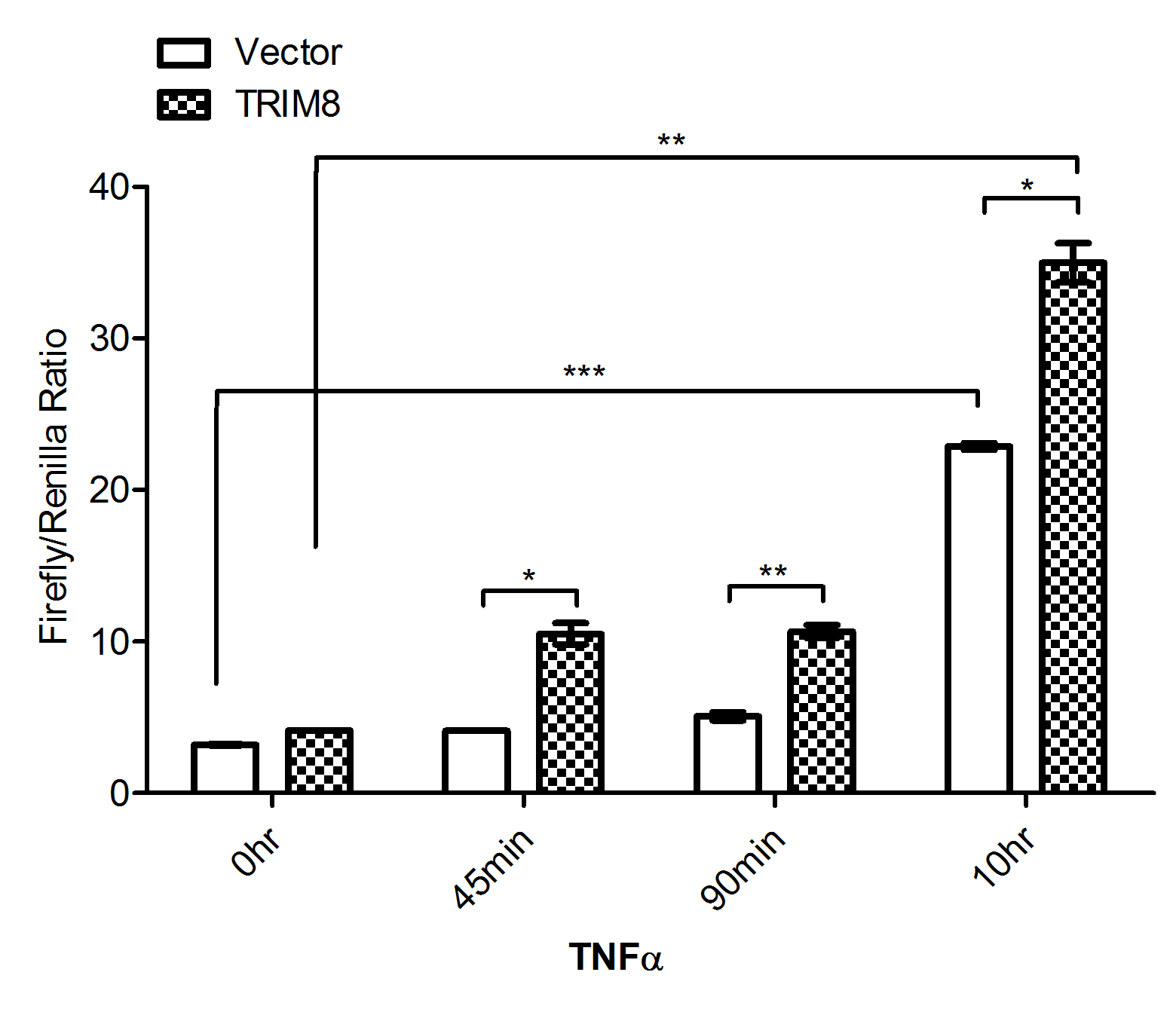

Supplement: Figure S1 — TRIM8 positively regulates TNF induced NF-κB. HEK293 cells were transfected with TRIM8 and vector; treated with TNFα (10 ng/ml) for different time interval and NF-κB activation measured by Dual Glo luciferase assay. Asterisk (*) indicates p value<0.05 for SEM of minimum three independent experiments. (TIF) [file pone.0048662.s001.tif]

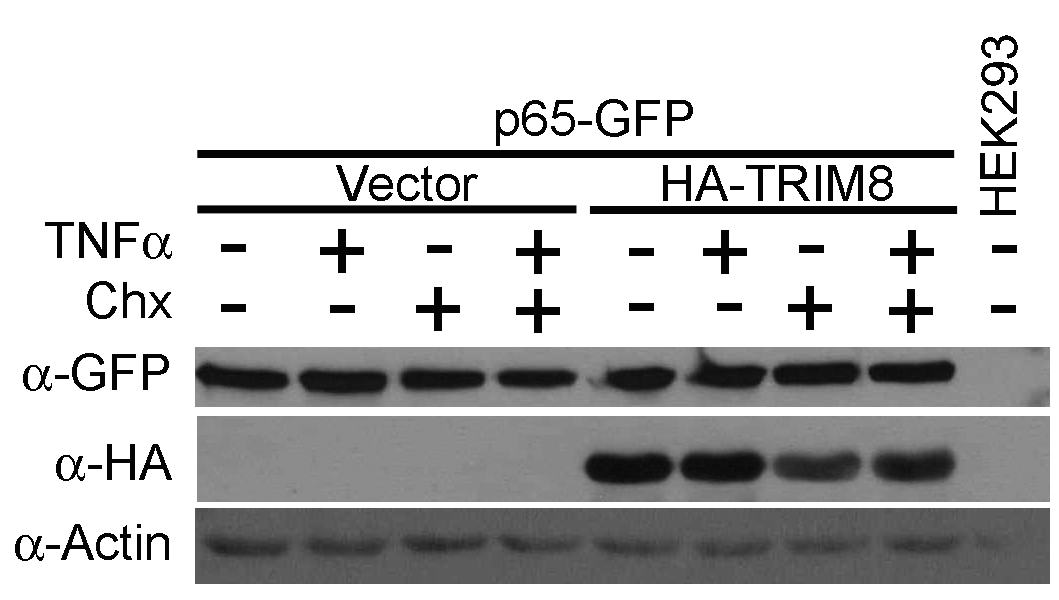

Supplement: Figure S2 — TRIM8 expression has no effect on p65 protein turnover. HEK293 cells were co-transfected with p65, TRIM8 and vector control. After 24 hours of transfection, the cells were treated with indicated chemical and incubated for 8 hours. The levels of different proteins were analyzed by western blotting using specific antibodies. HEK293 cell lysate was also loaded for negative control of HA-TRIM8 and p65-GFP. (TIF) [file pone.0048662.s002.tif]

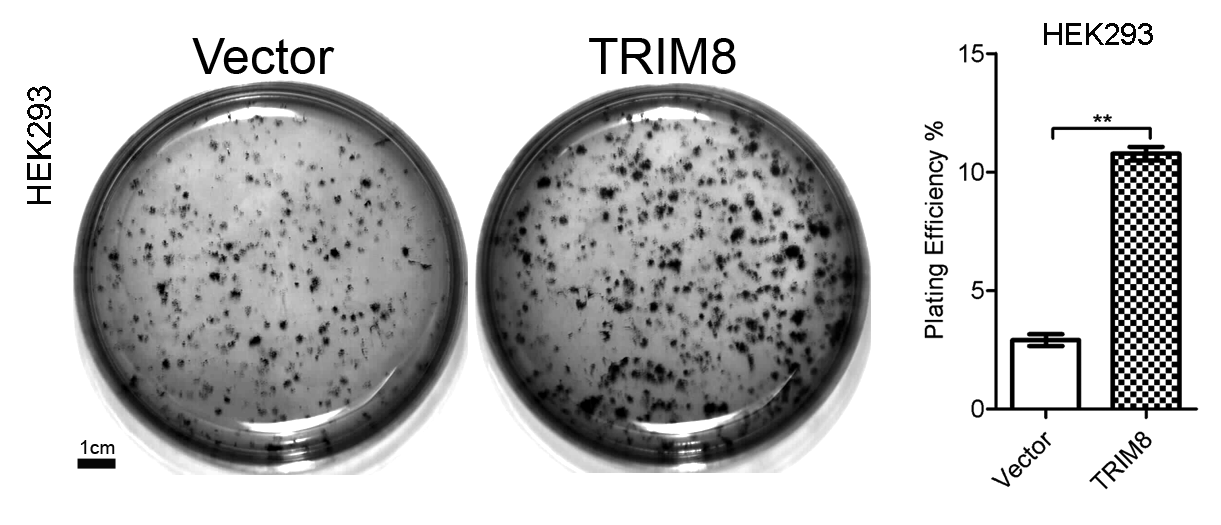

Supplement: Figure S3 — TRIM8 overexpression increases clonogenic ability of HEK293 cells. TRIM8 and vector were transfected in HEK293 cells and clonogenic ability analyzed as described in materials and method section. (TIF) [file pone.0048662.s003.tif]

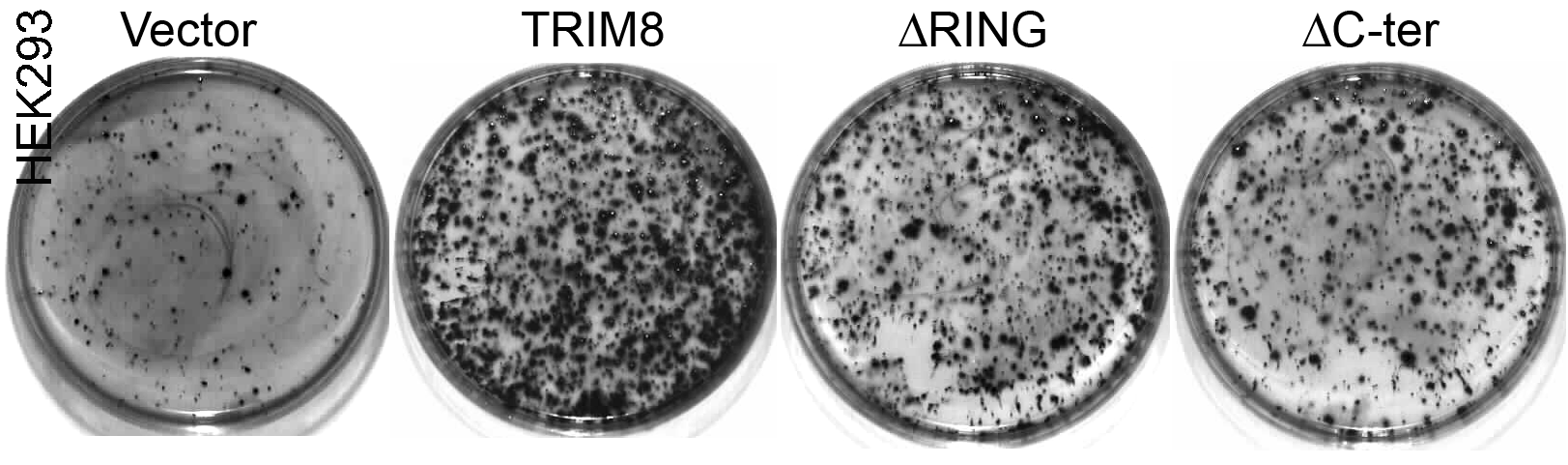

Supplement: Figure S4 — RING domain and C-terminal region of TRIM8 is essential in regulation of clonogenic ability of cells. FL-TRIM8, ΔRING, ΔC-ter and vector were transfected in HEK293 cells and clonogenic ability analyzed as described in materials and method section. (TIF) [file pone.0048662.s004.tif]
